# Extra-Axial Hematoma and Trimethoprim-Sulfamethoxazole Induced Aplastic Anemia: The Role of Hematological Diseases in Subdural and Epidural Hemorrhage

**DOI:** 10.1155/2015/374951

**Published:** 2015-06-23

**Authors:** Richard P. Menger, Rimal H. Dossani, Jai Deep Thakur, Frank Farokhi, Kevin Morrow, Bharat Guthikonda

**Affiliations:** Department of Neurosurgery, LSU Health Sciences Center-Shreveport, 1501 Kings Highway, P.O. Box 33932, Shreveport, LA 71130-3932, USA

## Abstract

*Objective and Importance*. To illustrate the development of spontaneous subdural hematoma secondary to aplastic anemia resulting from the administration of trimethoprim-sulfamethoxazole. This is the first report of trimethoprim-sulfamethoxazole potentiating coagulopathy leading to any form of intracranial hematoma. *Clinical Presentation*. A 62-year-old female developed a bone marrow biopsy confirmed diagnosis of aplastic anemia secondary to administration of trimethoprim-sulfamethoxazole following a canine bite. She then developed a course of waxing and waning mental status combined with headache and balance related falls. CT imaging of the head illustrated a 3.7 cm × 6.6 mm left frontal subdural hematoma combined with a 7.0 mm × 1.7 cm left temporal epidural hematoma. *Conclusion*. Aplastic anemia is a rare complication of the administration of trimethoprim-sulfamethoxazole. Thrombocytopenia, regardless of cause, is a risk factor for the development of spontaneous subdural hematoma. Given the lack of a significant traumatic mechanism, this subset of subdural hematoma is more suitable to conservative management.

## 1. Introduction

Intracranial presentation of bleeding secondary to hematologic abnormalities has a wide, striking, and often profound presentation [1]. Aplastic anemia (AA) represents a deficit across all three cell lineages [2]. This can occur de novo or secondary to a precipitating event such as radiation, infection, or medication [3]. In particular, aplastic anemia is a rare side effect of trimethoprim-sulfamethoxazole (TMP-SP), an antibiotic inhibiting bacterial nucleotide synthesis.

Intracranial hemorrhage (ICH) and subdural hematomas (SDH) are significant potential complications in patients with iatrogenic or de novo aplastic anemia. Intracranial bleeding affects up to 19.2% of patients with aplastic anemia. Intracranial hemorrhage is listed as the cause of death in up to 27.3% of patients with AA [4].

Numerous medications have been known to cause aplastic anemia and intracranial hemorrhage. However, there is no report of trimethoprim-sulfamethoxazole potentiating coagulopathy leading to any form of intracranial hemorrhage.

## 2. Case Presentation

62-year-old Caucasian female with a history of hyperlipidemia and osteopenia presented to an outpatient family medicine clinic after suffering a wild canine bite. The patient received standard rabies prophylaxis and subsequently underwent treatment with trimethoprim-sulfamethoxazole. 19 days later, the patient presented to an outside emergency room complaining of fatigue, dizziness, and multiple falls related to balance difficulty. After admission, the peripheral blood count revealed a white blood cell count of 1.2, suggesting a diagnosis of aplastic anemia. The bone marrow revealed hypoplastic bone marrow and hypoproliferation consistent with severe aplastic anemia. No metastatic carcinoma, viral inclusions, or granulomas were seen.

**Figure 1:**
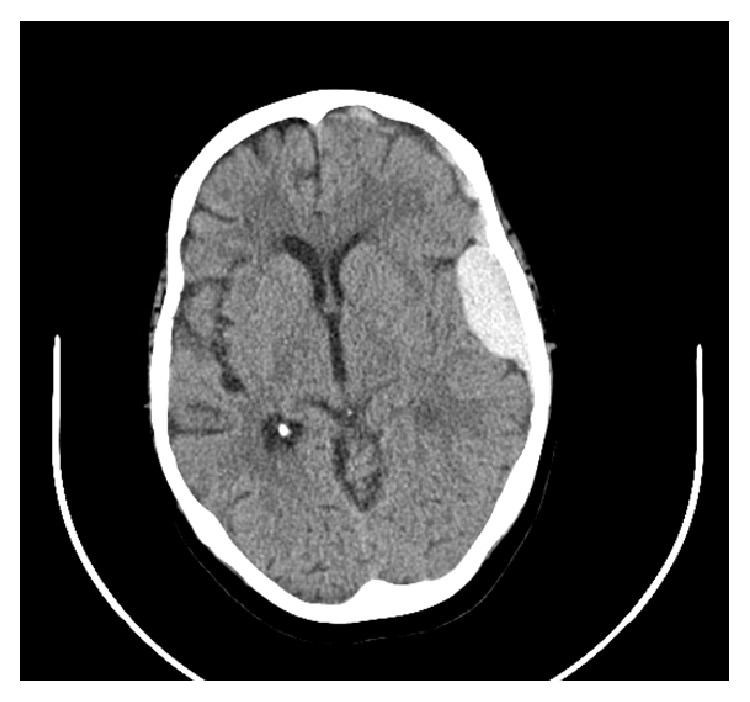
Initial axial CT head without contrast.

CT head revealed a 3.7 cm × 6.6 mm left frontal subdural hematoma, a 7.0 × 1.7 cm left temporal epidural hematoma, and left cerebral edema without midline shift (Figure 1). Upon neurosurgical consultation, the patient's examination showed the patient awake and fully oriented without any clinical neurological deficits. The patient was transferred to the intensive care unit for close monitoring. Platelets were transfused while hematology panels were sent for analysis. The patient's studies were negative for parvovirus, iron deficiency, and paroxysmal nocturnal hematuria but were notable for vitamin B12 deficiency. She was initiated on dexamethasone to induce demargination of cell lines, and was started on vitamin B12 replacement, and her antibiotic was changed from trimethoprim-sulfamethoxazole to vancomycin. Her platelets never reached the 100,000 benchmark despite transfusions, but her other cell lines improved. She was transferred out of the ICU and later discharged home in stable condition with a white count of 5.6, hemoglobin of 11.0, hematocrit of 32.7, and a platelet count of 52 K.

**Figure 2:**
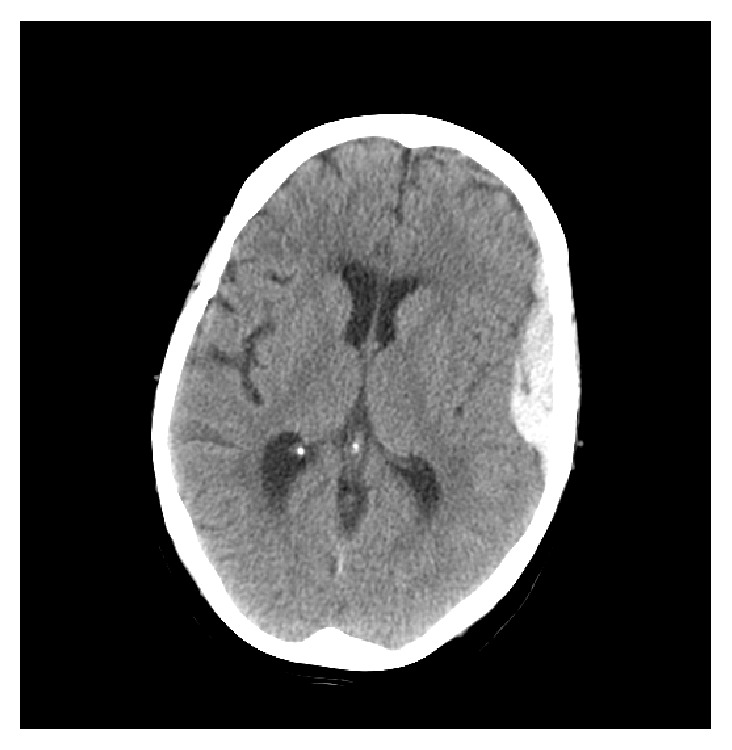
Repeat axial CT head without contrast.

Follow-up visit two days later revealed white count of 12.0, hemoglobin of 12.7, hematocrit of 37.0, and a platelet count of 117 K. She had no neurological deterioration and follow-up CT scan revealed reduction in the size of the epidural hematoma to 6.3 × 1.5 cm and the subdural hematoma to 2.5 cm × 4.4 mm (Figure 2). Final blood work was negative for HIV, hepatitis A, B, or C, bacterial infection, lupus, rheumatoid arthritis, and antiplatelet antibodies. The anemia was ultimately attributed to her trimethoprim-sulfamethoxazole use.

## 3. Discussion

Our case discusses the presentation and conservative management of a spontaneous SDH resulting secondary to aplastic anemia caused by TMP-SM, a widely used antimicrobial.

Historically, series have illustrated a conservative platform for monitoring of SDH as relatively incidental finding during chemotherapy. Pomeranz et al. monitored 471 bone marrow transplant patients finding subdural hematomas or intraparenchymal hemorrhages in 13/273 (4.7%) leukemia patients. No SDH were found in the aplastic anemia and beta-thalassaemia cohort. It is of note that no patients with leukemia suffered morbidity and mortality as a result of a SDH; patients with an intraparenchymal hematoma suffered a 100% mortality rate [5]. Specifically, the formation of SDH was linked to thrombocytopenia.

Sharma et al. illustrated the radiographic multifactorial progression of intracranial hemorrhage through aggressive MR imaging of 26 newly diagnosed AA patients over a six-month period. With an average platelet count of 11,200, 11.5% of patients in their series were seen to have asymptomatic cerebral microbleeds detected on gradient echo magnetic resonance imaging sequencing. No patients with cerebral microbleeds went on to develop symptomatic macrobleeds. Two separate patients developed symptomatic macrobleeds (greater than 1 cm) throughout the six months. Both of these developed de novo [3]. This was supplemented in Yamasaki et al. finding a varied presentation of intracranial bleeding secondary to aplastic anemia. Hemorrhages were notably varied through type, size, location, reoccurrence, and the presence of trauma [6].

One confounding factor in this patient's presentation is the presence of vitamin B12 deficiency. The patient presented with an initial B12 level of 146 pg/mL (normal range >150 pg/mL). At the time of consultation to the neurosurgical service, the patient's B12 level was >6000 pg/mL after receiving IV B12 injections. The patient did not have a pharmacological reason for this B12 deficiency; all rheumatologic or infectious etiologies were also investigated and subsequently ruled out.

Many disorders cause vitamin B12 deficiency, including lack of intrinsic factor production by the parietal cells of the stomach and malabsorption due to bowel resection or inflammatory bowel disease [7]. Serum B12 levels are used to make the diagnosis of B12 deficiency [8]. However, serum B12 levels may not be reliable in the setting of myeloproliferative disorders or folate deficiency. B12 deficiency can cause folate deficiency with shunting of tetrahydrofolate (THF) to nonhydrolyzable methyl-THF [7]. Specifically the patient was noted to have a folate level of 7.9 ng/mL (normal range 8.7–55.4 ng/mL) and was subsequently initiated on oral folate replacement.

Levels of B12 >300 ng/L exclude B12 deficiency and levels <150 ng/L are considered to be diagnostic of B12 deficiency. For levels between 150 and 300 ng/L, levels of methylmalonic acid and homocysteine are more sensitive and specific for diagnosis of B12 deficiency [7]. In our patient, methylmalonic acid level was 235 nmol/L and homocysteine level was 7.7 *μ*mol/mL, both within normal limits. In both children and adults, vitamin B12 deficiency is associated with pancytopenia, megaloblastic anemia, splenomegaly, and retinopathy [8]. In the literature, platelet count in patients with B12 deficiency is in the order of 80–100,000, although lower platelet counts have been documented [8].

The etiology of our patient's B12 deficiency remains elusive and may have been dietary in origin. In this patient, the more likely cause of intracranial bleed was folate deficiency secondary to TMP-SM administration. This is due to profound thrombocytopenia (platelet count of 12,000) combined with the rapid improvement in platelet count with the cessation of TMP-SM administration. Platelet levels take up to a month to normalize in the setting of B12 replacement [7]. This patient improved within five days after the discontinuation of TMP-SM and the addition of folate. What is most likely is that an already hypocobalaminemic (and therefore folate deficient) patient had an acute exacerbation of folate deficiency due to further disruption of folate production secondary to the addition of TMP-SM.

The presentation of our patient is typical of the multifactorial and cryptic nature of intracranial bleeding secondary to hematological disorders. Hematological disorders have been historically described to cause myriad of intracranial hemorrhagic disorders [9]. Although a rare event, ICH secondary to hematological disorders requires vigilant neurological attention to monitor bleed progression and to prevent devastating outcomes in this subset of patients. Although the pathophysiology of ICH in hematological imbalance is not understood well, disseminated intravascular coagulation, alteration of membrane permeability by leukemic cells, leukostasis, hypoxic vasodilatation, and clinically significant thrombocytopenia/platelet dysfunction can explain their tenacity to debilitating hemorrhages [10].

Lee and Kim found that the mean platelet count of patients with idiopathic thrombocytopenic purpura (ITP) who developed SDH was 26,700 while those who developed ICH was 14,300 [11]. All patients with subdural hematoma subsequently improved without surgical intervention. However, in Lee and Kim the mortality of ITP related intracranial hemorrhage was identical to those of spontaneous ICH. Furthermore, it is specifically noted that formation of the SDH is correlated to the thrombocytopenia but not the enlargement of the hematoma [12]. Although there is no reported direct trauma in our case or in the literature, it is highly likely that microtrauma is related to the development of SDH in the face of thrombocytopenia.

Management of thrombocytopenia in the setting of aplastic anemia includes aggressive platelet infusion; however, consensus regarding the optimal value is lacking in the literature. Target for adequate platelet function in such setting varies from 20,000 *μ*L to 50,000 *μ*L [3]. This is based on understanding that the pathophysiology of the bleed in such patients varies due to a continuum of dysfunctional hemostatic events as discussed by González-Duarte et al. Isolated value of platelet count above 100,000 *μ*L is not consistently reproducible in every patient with aplastic anemia to prevent hematoma evolution. Although neurosurgical literature supports platelets more 100,000 *μ*L for preventing evolution of ICB, such target may not be amenable in the setting of aplastic anemia (especially in congenitally acquired cases). We used goal of 50,000 *μ*L in our patient with successful prevention of bleed expansion with 50,000 *μ*L being on the relatively higher limit published in the literature for restoring the platelet function.

## 4. Conclusion

Thrombocytopenia, regardless of cause, is a risk factor for the development of spontaneous subdural hematoma. This is in contrast acute traumatic extra-axial hematoma associated with an obvious traumatic event; that patient subset has significant morbidity. Detailed history and physical examination is critical to the management of subdural hematoma in the context of hematological dysfunction.
